# Validation of Contrast-Enhanced Mammography as Breast Imaging Modality Compared to Standard Mammography and Digital Breast Tomosynthesis

**DOI:** 10.3390/diagnostics14141575

**Published:** 2024-07-21

**Authors:** Nina Bartolović, Ana Car Peterko, Manuela Avirović, Doris Šegota Ritoša, Emina Grgurević Dujmić, Petra Valković Zujić

**Affiliations:** 1 Department of Diagnostic and Interventional Radiology, Clinical Hospital Centre Rijeka, Kresimirova 42, 51000 Rijeka, Croatia; 2 Department of General Surgery and Surgical Oncology, Clinical Hospital Centre Rijeka, Kresimirova 42, 51000 Rijeka, Croatia; 3 Department of Pathology, Faculty of Medicine, University of Rijeka, Brace Branchetta 20, 51000 Rijeka, Croatia; 4 Department of Pathology, Clinical Hospital Centre Rijeka, Kresimirova 42, 51000 Rijeka, Croatia; 5 Medical Physics and Radiation Protection Department, Clinical Hospital Centre Rijeka, 51000 Rijeka, Croatia; 6 Community Health Centre Primorsko-Goranska County, Kresimirova 52A, 51000 Rijeka, Croatia; 7 Department of Radiology, Faculty of Medicine, University of Rijeka, Brace Branchetta 20, 51000 Rijeka, Croatia

**Keywords:** breast cancer, contrast media, digital breast tomosynthesis, mammography

## Abstract

Contrast-enhanced mammography (CEM) is a relatively new imaging technique that allows morphologic, anatomic and functional imaging of the breast. The aim of our study was to validate contrast-enhanced mammography (CEM) compared to mammography (MMG) and digital breast tomosynthesis (DBT) in daily clinical practice. This retrospective study included 316 consecutive patients who underwent MMG, DBT and CEM at the Centre for Prevention and Diagnosis of Chronic Diseases of Primorsko-goranska County. Two breast radiologists independently analyzed the image data, without available anamnestic information and without the possibility of comparison with previous images, to determine the presence of suspicious lesions and their morphological features according to the established criteria of the Breast Imaging Reporting and Data System (BI-RADS) lexicon. The diagnostic value of MMG, DBT and CEM was assessed by ROC analysis. The interobserver agreement was excellent. CEM showed higher diagnostic accuracy in terms of sensitivity and specificity compared to MMG and DBT, the reporting time for CEM was significantly shorter, and CEM findings resulted in a significantly lower proportion of equivocal findings (BI-RADS 0), suggesting fewer additional procedures. In conclusion, CEM achieves high diagnostic accuracy while maintaining simplicity, reproducibility and applicability in complex clinical settings.

## 1. Introduction

Contrast-enhanced mammography (CEM) is a relatively new and promising imaging modality that combines the morphologic information of mammography (MMG) and digital breast tomosynthesis (DBT) with the functional advantages of magnetic resonance imaging (MRI) [1].

Although MMG remains the first and most important imaging modality in breast radiology, it has limited accuracy in women with dense breasts, who make up slightly less than half of the screening population [2]. The sensitivity of MMG decreases significantly with increasing density of the breast parenchyma, so the sensitivity is lower in woman with dense breasts (between 62.9 and 72.9%) than in women with fatty breasts (81.5 to 87.0%) [1,3,4].

Digital breast tomosynthesis allows each individual layer to be analyzed, reducing the overlapping effect of glandular tissue, which is crucial in dense breasts. This improves the visualization of lesions and increases the sensitivity of MMG [4,5].

Currently, MRI is the imaging modality with the highest sensitivity for the detection of breast cancer, between 79 and 98%, and is indispensable in breast imaging practice. It is used to detect and adequately characterize the lesion and to determine the extent of the disease by assessing the ipsilateral and contralateral breast. However, its widespread use is limited by factors such as high cost, long image acquisition time, limited availability, claustrophobia and specific contraindications, e.g., in patients with cardiac pacemakers, aneurysms clips and other metallic implants [5,6]. Therefore, alternative diagnostic methods must be considered. 

Contrast-enhanced mammography is a relatively new imaging technique that allows morphological, anatomical and functional imaging of the breast using the same principle of physiological contrast enhancement as breast MRI, but with intravenous administration of iodinated contrast agents. It outperforms mammography in sensitivity (95% vs. 84%; *p* < 0.025) and specificity (81% vs. 63; *p* < 0.025) [7] and has a comparable sensitivity to MRI in the visualization and localization of pathological lesions (91% vs. 97%; *p* < 0.01), with statistically equivalent specificity (74% and 69%; *p* = 0.09), respectively [8,9]. Compared to MRI, it has numerous advantages: lower costs, shorter image acquisition time, better acceptance and easier tolerance by patients and the major advantage of widespread availability [9]. It is also a preferred imaging modality for patients with claustrophobia or specific contraindications for MRI. In addition to the contrast enhancement of breast lesions, CEM can also visualize pathological calcifications that are not visible on MRI. However, CEM also has disadvantages, including radiation exposure, the lack of three-dimensional visualization, the need for compression, the use of iodinated contrast agents and the lack of visualization of the axilla [9,10]. 

Although recent studies have highlighted the diagnostic capabilities of CEM, which have been increasingly recognized in recent years, the optimal applications of CEM compared to other breast imaging modalities remain unclear, necessitating further research to fully establish its role in clinical practice.

Several critical gaps in the current state of research need to be addressed. One of the primary areas where further research is required involves the diagnostic accuracy of CEM across diverse patient populations. Studies have demonstrated CEM’s effectiveness in specific cohorts [10,11,12], but its performance in broader demographic groups, including variations in breast density, age and genetic risk factors, remains underexplored. Understanding how CEM performs across these different subgroups is crucial for determining its generalizability and applicability in routine clinical settings. 

Recent research efforts have focused on evaluating the diagnostic capabilities of CEM in specific clinical contexts. For instance, studies have examined the use of CEM for pre-surgical staging of newly diagnosed breast cancers and its effectiveness in predicting breast cancer in symptomatic patients [1,12]. A clinical trial on CEM highlighted its potential as a reliable imaging modality for breast cancer detection, offering a viable alternative for patients with contraindications for MRI or those who cannot tolerate MRI procedures [8,9]. Additionally, while CEM has shown promise as an alternative to MRI, the exact scenarios in which CEM can effectively replace or complement MRI are not fully delineated. Direct comparative studies assessing both modalities in various diagnostic contexts are necessary to clarify their respective roles and benefits. Such comparisons would help define specific clinical situations where CEM may offer advantages over MRI, including considerations of cost, availability and patient comfort. 

Another significant gap in the literature pertains to the cost-effectiveness and accessibility of CEM. Although CEM is generally more affordable and quicker than MRI [8,9,13], comprehensive economic analyses evaluating the cost–benefit ratio of incorporating CEM into standard screening protocols are limited. Understanding the financial implications of widespread CEM adoption, especially in resource-limited settings, is essential for health policy planning and decision making. 

Furthermore, the impact of CEM on clinical decision making and patient management requires deeper investigation. While initial studies suggest that CEM can reduce the number of unnecessary biopsies and follow-up procedures by providing clearer differentiation between benign and malignant lesions [1,12,13], more evidence is needed to confirm these benefits and understand their long-term implications. Longitudinal studies tracking patient outcomes, such as recurrence rates, survival and quality of life, are particularly valuable for assessing the sustained benefits and potential risks associated with CEM.

This study aimed to investigate and determine the diagnostic value of CEM compared to MMG and DBT in daily clinical practice, thus contributing to the growing body of evidence supporting CEM’s use, particularly as a stand-alone breast imaging technique. Furthermore, by evaluating its impact on patient management, this study shows the potential of CEM to improve clinical practice by enabling faster and more accurate breast cancer diagnosis. 

## 2. Materials and Methods

### 2.1. Study Population

After institutional review board approval and a waiver of informed consent, a single-center retrospective study was conducted. 

We searched the database for consecutive patients who underwent MMG, DBT and CEM at the Centre for Prevention and Diagnosis of Chronic Diseases of Primorsko-goranska County between August 2021 and March 2024. Included in the study were patients older than 18 years and patients whose MMG and DBT reports were classified as incomplete (BI-RADS 0), suspicious (BI-RADS 4) and highly suggestive of malignancy (BI-RADS 5) [14], i.e., patients referred for CEM. The exclusion criteria were technically inadequate CEM examinations (inadequate positioning (n = 2), contrast extravasation (n = 1), failed subtraction images (n = 0) and patients who underwent additional procedures outside the Clinical Hospital Centre Rijeka (n = 21). The final study group consisted of 316 patients (median age 64 years; range 37–88 years). 

**Figure 1:**
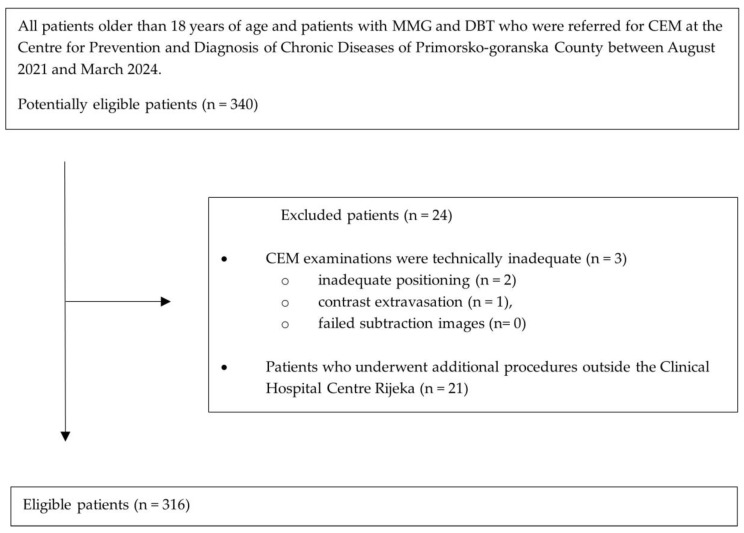
Standards of Reporting of Diagnostic Accuracy (STARD 2015) flow chart of included patients with MMG, DBT and CEM. MMG, mammography; DBT, digital breast tomosynthesis; CEM, contrast-enhanced mammography.

The flow chart of patients included in the study is based on the Standards for Reporting of Diagnostic Accuracy (STARD 2015) guidelines (Figure 1).

Patient characteristics, including age, gender, history of previous breast biopsy or surgery were obtained from the institutions’ medical records. 

Patients whose CEM reports were categorized as BI-RADS 4 or BI-RADS 5 and included the recommendation for tissue biopsy subsequently underwent the procedure at the Clinical Hospital Center Rijeka (CHCR). The data on the date and type of the procedure and the histopathological findings were taken from the medical records of the CHCR.

### 2.2. CEM Protocol and Image Reconstruction

All MMG, DBT and CEM examinations were performed with a digital mammography unit (Siemens Mammomat Revelation, Siemens Healthineers, Erlangen, Germany). All patients whose MMG and DBT reports were classified as BI-RADS 0, BI-RADS 4 and BI-RADS 5 were referred for CEM.

Before the CEM examination, the patients were informed about the examination protocol and possible side effects of the iodine-containing contrast media and signed the institutional consent forms. After collecting the anamnestic data and assessing the renal function values, the radiologist placed the intravenous catheter in the antecubital fossa. A dose of 1.5 mL/kg of iodinated contrast medium (300–370 mgI/mL) was administered via an injector at a rate of 2–3 mL/s, followed by a saline flush of 20 mL at the same flow rate to increase the release of the contrast medium into the tissue and improve image quality. Venous access was maintained until the end of the examination and the patient was monitored throughout the procedure to enable immediate treatment of any adverse reactions to the iodinated contrast agent. The patient was positioned for mammography and imaging began two minutes after injection. Imaging included classic craniocaudal (CC) and mediolateral oblique (MLO) projections for both breasts, at low and high energy. We always started with the breast where the suspicious finding was located to emphasize early enhancement and avoid false negative findings due to early washout; the contralateral breast was then imaged. If enhancement was observed on the suspicious side, a further projection was performed after 8 min to qualitatively assess the kinetics of enhancement and determine the likelihood of malignancy. The examination was completed after eight to ten minutes. 

Low-energy X-rays were taken at the same kVp as for digital mammography, i.e., 26–33 kVp, and with the same rhodium or tungsten filter. High-energy images, on the other hand, were taken at 49 kVp, i.e., higher kVp values, using a titanium and tungsten filter. The recombined images were generated by removing background glandular tissue and sent to the picture archiving and communication system (PACS) together with the low-energy images [7].

### 2.3. Image Analysis

**Figure 2:**
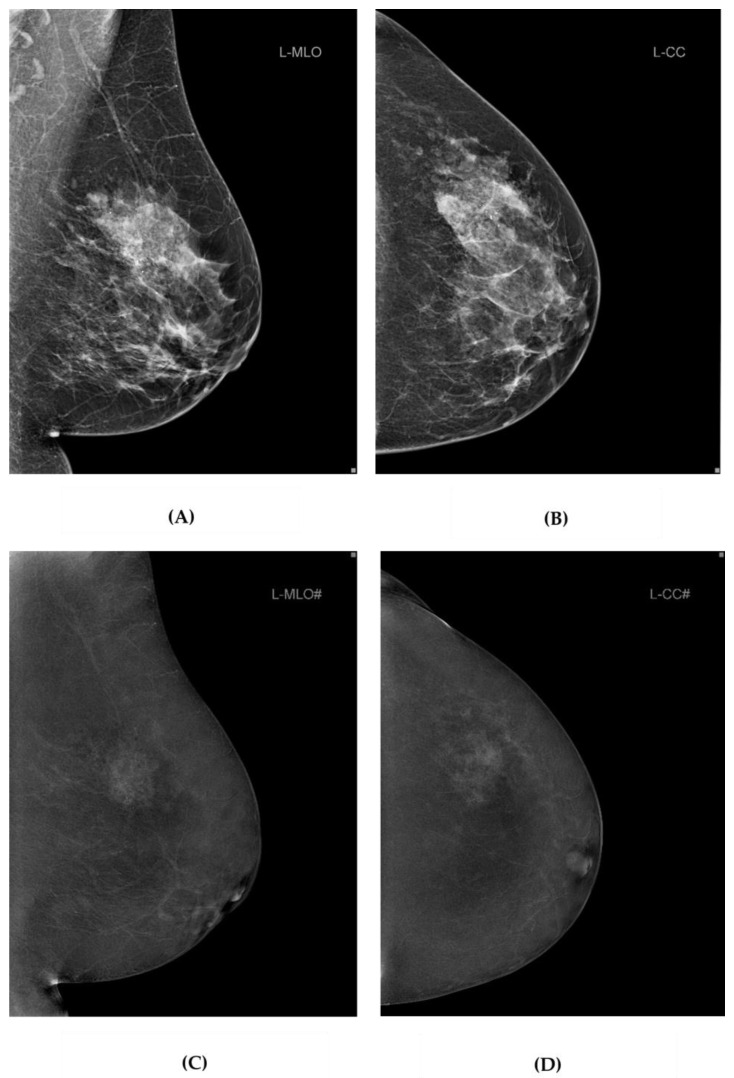
MMG and DBT and CEM image data from our study. Standard MMG (**A**,**B**), and CEM (**C**,**D**) projections of the left breast of a 63-year-old female participant with breast cancer in UOQ. MMG, mammography; DBT, digital breast tomosynthesis; CEM, contrast-enhanced mammography; UOQ, upper outer quadrant.

Two radiologists with 14 and 5 years of experience in breast radiology independently analyzed MMG and DBT image data as well as CEM image data (Figure 2). 

The researchers were blinded to previous radiology reports, anamnestic information, medical history and clinical data. Furthermore, there was no possibility of comparison with previous images. Images were analyzed at two different time points three months apart to minimize memory bias (recall errors). The MMG and DBT image data were analyzed first, and the CEM image data were analyzed after the designated time interval.

Consistent measurement of the time taken to analyze the MMG and DBT and CEM images was achieved by activating a stopwatch at the beginning of the study and deactivating it when reviewing the written report. 

The images were analyzed on a dedicated high-resolution workstation (Nio Gray 5.8MP, MDNG-6211, Barco, Kortrijk, Belgium). 

The aim was to determine the presence of a suspicious lesion in the breast and its morphological characteristics according to the established criteria of the Breast Imaging Reporting and Data (BI-RADS) lexicon [14]. Only one suspicious lesion was considered, and the most suspicious lesion was selected if several were detected in the analyzed image data.

### 2.4. Statistical Analysis

Statistical analysis of the data was performed with MedCalc version 22.021 (MedCalc Software, MariaKerke, Belgium). The normality of the distribution was checked with the Kolmogorov–Smirnov test. Categorical data are presented with absolute and relative values, while numerical data are presented with median and 5th and 95th percentiles. Age is presented with median and absolute range. 

The interrater agreement between two different researchers in scoring the BI-RADS category was calculated using the interclass correlation coefficient (ICC), a grading system developed by Koo TK and Li MY, and the scores were determined as follows: >0.50, poor; 0.50–0.75, moderate; 0.75–0.90, good; >0.9, excellent agreement [15]. Due to the excellent correlation between the two researchers, the data set obtained from only one researcher (researcher 1) was used for further analysis. 

Comparisons of BI-RADS categories for MMG and DBT as well as CEM reports were calculated using the Hi-square test and a comparison of proportions as a post hoc test, while differences in time criterion were calculated using the Wilcoxon paired samples test. 

Receiver operating characteristic curve–area under the curve (ROC-AUC) analysis was used to calculate the sensitivity and specificity of the MMG, DBT and CEM examinations. The efficiency of a criterion was calculated by the area under the ROC curve (area under the curve, AUC), and these values were also represented by the ROC curve. The comparison of the ROC curve was calculated between the MMG and DBT as well as the CEM examination to determine better sensitivity and specificity of the method. 

All statistical values were considered significant if the *p*-value (*p*) was <0.05.

## 3. Results

### 3.1. Patient Characteristics

A total of 316 women underwent MMG, DBT and CEM examinations (Figure 1). The median age of the study participants was 64 years (range 37–88 years). The characteristics of participants are listed in Table 1. 

### 3.2. Image Analysis and BI-RADS Categories

To assess the reliability and interrater agreement of the researchers’ observations with the established BI-RADS lexicon criteria, we calculated the interclass correlation coefficient for both examinations, as shown in Table 2.

Interrater agreements were satisfactory for both measures. Agreement between researchers 1 and 2 was moderate for MMG and DBT (r = 0.73, *p* < 0.05) and excellent for CEM (r = 0.98, *p* < 0.05). Where no comparison between researchers is presented, we only used the data set of one of the observers in the sample (researcher 1) for the following statistical analysis.

**Figure 3:**
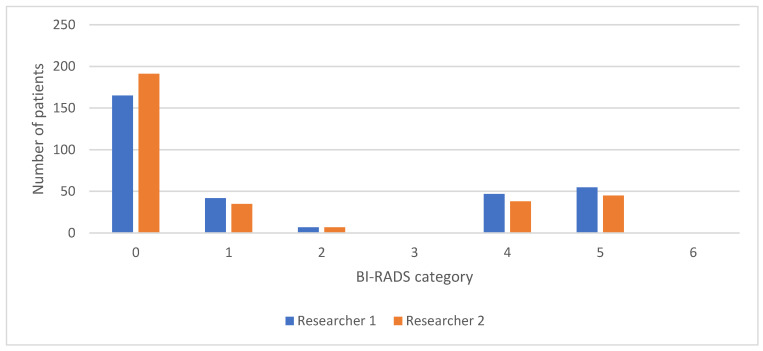
Comparison of BI-RADS categories for MMG and DBT reports between two researchers. BI-RADS, Breast Imaging Reporting and Data System; MMG, mammography; DBT, digital breast tomosynthesis.

**Figure 4:**
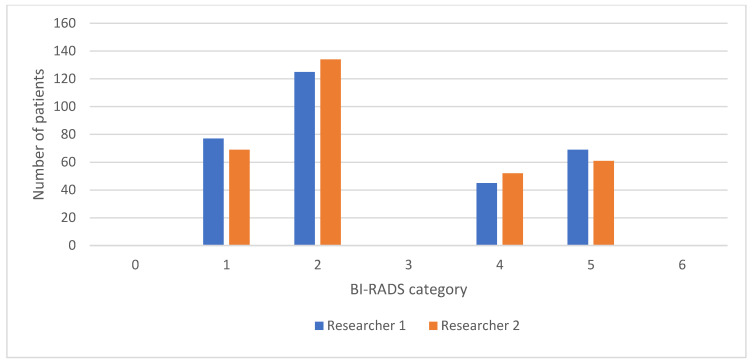
Comparison of BI-RADS categories for CEM reports between two researchers. BI-RADS, Breast Imaging Reporting and Data System; CEM, contrast-enhanced mammography.

The interrater agreement is presented as a comparison of the frequencies of BI-RADS categories for MMG and DBT between the two researchers, as shown in Figure 3, while the comparison of the frequencies of BI-RADS categories for CEM between the two researchers is shown in Figure 4.

The frequencies and differences of the BI-RADS categories for MMG and DBT reports and for CEM reports are shown in Table 3.

MMG and DBT were categorized as incomplete (BI-RADS 0) reports in more than half of the participants (52.2%), as opposed to zero incomplete (BI-RADS 0) CEM reports.

**Figure 5:**
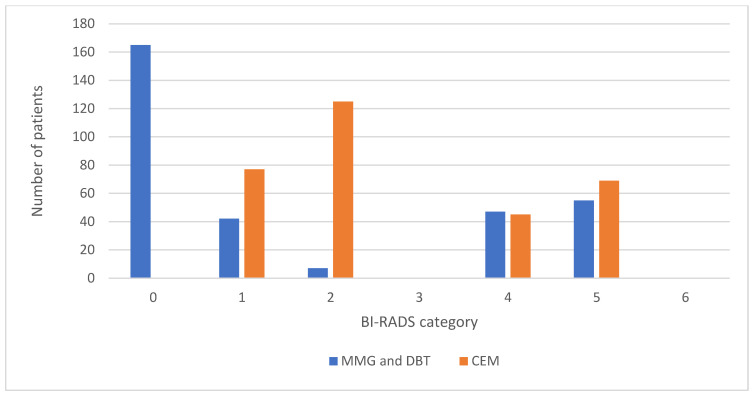
Comparison of BI-RADS categories for MMG and DBT and CEM reports—researcher 1. BI-RADS, Breast Imaging Reporting and Data System; MMG, mammography; DBT, digital breast tomosynthesis; CEM, contrast-enhanced mammography.

**Figure 6:**
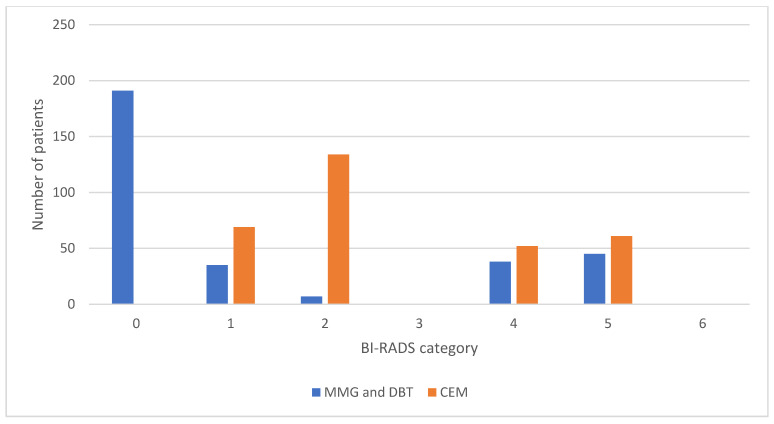
Comparison of BI-RADS categories for MMG and DBT and CEM reports—researcher 2. BI-RADS, Breast Imaging Reporting and Data System; MMG, mammography; DBT, digital breast tomosynthesis; CEM, contrast-enhanced mammography.

There was also a statistical difference in the BI-RADS 2 category: there were seven participants (2.2%) with MMG and DBT reports categorized in the BI-RADS 2 category compared to 125 participants (39.6%) with CEM reports categorized in the same BI-RADS category (*p* = 0.048). There were no other statistical differences (all *p* > 0.05) between the two studies for other BI-RADS categories. The comparison of the frequencies of BI-RADS categories for MMG and DBT and CEM reports is shown in Figure 5 and Figure 6. Figure 5 shows the frequencies observed by researcher 1 (as in Table 3), while Figure 6 shows the frequencies observed by researcher 2. 

Both figures show a high number of incomplete (BI-RADS 0) categories for MMG and DBT reports and zero incomplete (BI-RADS 0) categories for CEM reports. No observations were made by either researcher for the “probably benign” (BI-RADS 3) and “biopsy proven” (BI-RADS 6) categories (Figure 5 and Figure 6).

### 3.3. Reporting Time

As shown in Table 4, the median reporting time was statistically lower for CEM compared to MMG and DBT (*p* < 0.0001).

The shortest reporting time for MMG and DBT examinations was 51 s and the longest 186 s, with a median of 96 s per report, which was statistically higher than the reporting time for CEM examinations, with the shortest reporting time of only 15 s and the longest of 183 s, with a median of 66 s per report (Z = −12.17, *p* < 0.0001).

### 3.4. Receiver Operating Characteristic Curve Analysis 

ROC-AUC analysis was used to determine the sensitivity and specificity of MMG and DBT, as well as CEM, and to compare the two methods of breast imaging.

**Figure 7:**
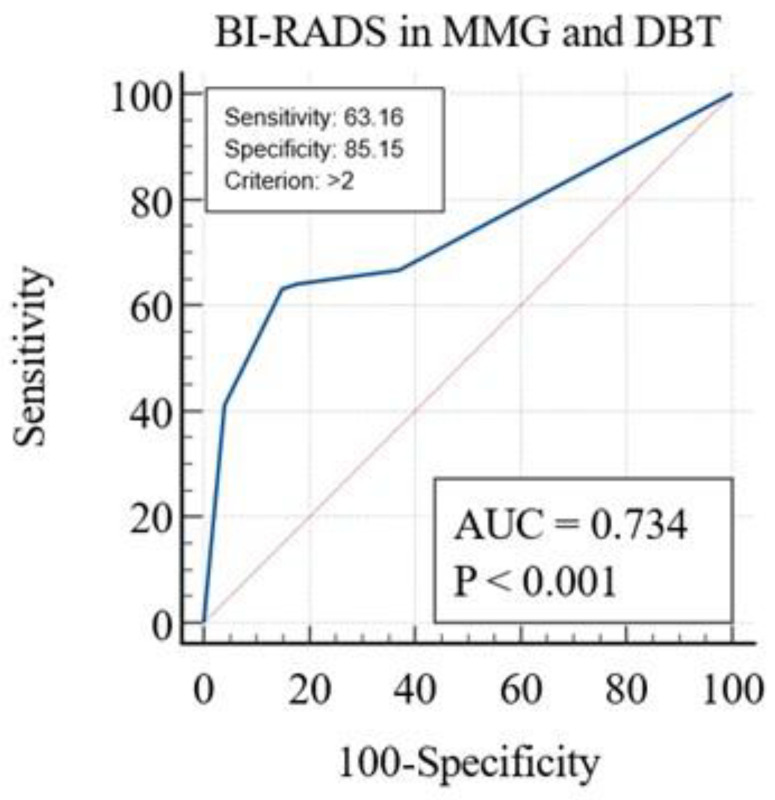
Receiver operating characteristic curve analysis for MMG and DBT. MMG, mammography; DBT, digital breast tomosynthesis; CEM, contrast-enhanced mammography.

MMG and DBT had a sensitivity of 63.16% and a specificity of 85.15% (AUC = 0.73, *p* < 0.001), as shown in Figure 7. The cut-off value was set to the BI-RADS 2 category.

**Figure 8:**
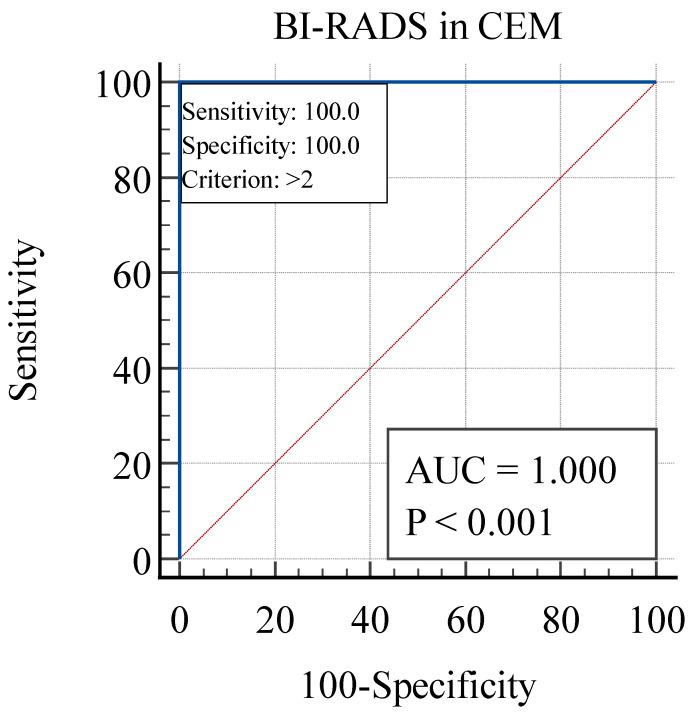
Receiver operating characteristic curve analysis for CEM. CEM, contrast-enhanced mammography.

Opposite to MMG and DBT, CEM had a sensitivity of 100% and specificity of 100% (AUC = 1.0, *p* < 0.001), as shown in Figure 8. The cut-off value was set to the BI-RADS 2 category.

As a result of ROC-AUC analyses, CEM has statistically better characteristics than MMG and DBT (Z = 8.78, *p* < 0.001). CEM has a better AUC (1.0 vs. 0.73), a higher sensitivity (100 vs. 63.16) and a higher specificity (100 vs. 85.15) compared to MMG and DBT.

## 4. Discussion

Contrast-enhanced mammography had a sensitivity of 100% (AUC = 1.0, *p* < 0.001), which was significantly higher than MMG and DBT with a sensitivity of 63.16% (AUC = 0.73, *p* < 0.001). CEM also had a significantly higher specificity (100%, AUC = 1.0, *p* < 0.001) than MMG and DBT (85.15%, AUC = 0.73, *p* < 0.001). Thus, CEM improved the sensitivity of MMG and DBT without affecting specificity. The sensitivity of CEM reported by previous authors ranged from 63.5 to 100%. A study published by Lusczynska et al. compared MMG, CEM and ultrasound in 116 patients with 137 lesions and reported 100% sensitivity of CEM, 10% higher than MMG (*p*-value < 0.004) and 8% higher than ultrasound (*p*-value < 0.01) [16].

Three studies involving 507 women in a post-screening study showed that the sensitivity of CEM is between 93% and 100% and the specificity between 63% and 88% [17,18,19]. Studies suggest that CEM is superior to conventional MMG and DBT, it increases diagnostic accuracy, and it is an excellent problem-solving tool for recalls from screening programs. A study by Cozzi et al. included 207 patients recalled from screening mammography and examined with CEM and reported a sensitivity of 94% and a specificity of 66% for malignancy detection, with a 16% biopsy rate reduction compared to standard screening [20]. Nicosia et al. point out that CEM has better specificity for benign cysts compared to MMG because it does not show internal enrichment, which could reduce screening recall rates [21]. Thus, CEM may increase both sensitivity and specificity in the diagnostic evaluation of screening recalls compared to standard evaluation, resulting in lower biopsy rates.

Another study conducted by Lobbes et al. confirms the above thesis that CEM is an excellent problem-solving tool for inconclusive findings in screening mammography, especially for reducing the number of false positive recalls. In addition, CEM is associated with a high NPV, suggesting that negative CEM can be used to rule out malignancy and the need for short-term follow-up in these women [16]. In addition, a feasibility study by Zuley et al. showed that CEM significantly reduced the false positive rate (FPR) (*p* = 0.017) and significantly increased the true positive rate (TPR) (*p* = 0.019) for BI-RADS 4 soft tissue lesions compared to FFDM/DBT. Even when combined with ultrasound, the TPR of FFDM/DBT did not reach that of CEM, while the FPR increased significantly. These results suggest that CEM is probably more accurate than the combination of FFDM/DBT/US. Furthermore, performing an additional ultrasound examination after a negative CEM finding is questionable as it increases the risk of detecting false positive lesions without any real improvement in cancer detection [22]. In line with these studies and the results of the study conducted by Lalji et al. [19], no follow-up on final CEM findings categorized as BI-RADS 1 and BI-RADS 2 was performed in our study. Furthermore, our imaging strategy in these cases is also in line with the NHSBSP clinical guidelines for breast cancer screening assessment [23]. This strategy is safe, and the likelihood of breast cancer being missed is minimal.

Our results are consistent with the prospective study by Sudhir et al. in which CEM was compared with MMG and ultrasound assessment of 166 breast lesions in 130 symptomatic patients. The study showed better sensitivity for cancer detection in dense breasts with CEM (97%) compared to MMG (76%). The sensitivity was also higher than the reported sensitivity of 83% for DBT [1]. This study suggests that CEM could be used as a stand-alone imaging modality in symptomatic patients, particularly in dense breasts. A multi-reader study by Girometti et al. on preoperative staging of 78 patients with 100 lesions, comparing CEM with diagnostic MMG plus DBT, showed a higher detection rate of additional malignant sites on CEM, especially in dense breasts [24]. This study suggests that the use of CEM in preoperative planning will provide similar benefits to MRI and better assessment of disease extent than MMG and DBT.

The results of our study include a notable discrepancy in the number of reports categorized as BI-RADS 0 between MMG and DBT (165 reports, 52.2%) and CEM (0 reports, 0%), with moderate interrater agreement between researchers 1 and 2 for MMG and DBT (r = 0.73, *p* < 0.05) and excellent interrater agreement for CEM (r = 0.98, *p* < 0.05) and the statistical difference in the BI-RADS 2 category with seven MMG and DBT findings (2.2%) versus 125 CEM findings (39.6%) categorized as BI-RADS 2. These results are consistent with previous studies and support the use of CEM as a straightforward problem-solving method for equivocal MMG and DBT findings, providing more confidence to radiologists. It is desirable for any diagnostic method to be as simple as possible without compromising diagnostic accuracy, and CEM has demonstrated all these qualities when compared to mammography and tomosynthesis. Furthermore, this reduction in reports categorized as BI-RADS 0 can decrease the need for additional imaging and follow-up procedures, thereby minimizing patient anxiety, reducing healthcare costs and streamlining the diagnostic process.

The study reports satisfactory interrater agreements for both measures: moderate agreement for MMG and DBT (r = 0.73, *p* < 0.05) compared to excellent agreement for CEM (r = 0.98, *p* < 0.05). The excellent interrater agreement for CEM, or in other words, the high level of consistency among different radiologists interpreting CEM, underscores its reliability and potential for widespread clinical adoption, thus ensuring high-quality patient care and making CEM a dependable tool in clinical practice.

This is confirmed by two studies. In a multi-reader study by Lalji et al. [19], seven radiologists and three residents (representing three levels of experience) evaluated 199 cases and showed that specificity and diagnostic performance improved significantly with CEM compared to FFDM, regardless of experience level. The sensitivity scores of residents (96.6%) and less-experienced CEM readers (95.9%) were comparable to those of experienced readers (97.6%). These results suggest that novice CEM readers can achieve the same proficiency as experienced radiologists. This is also supported by another study in which inexperienced high school students, after a brief introduction to breast cancer and CEM, evaluated the cases from a study by Lalji et al. and immediately achieved a sensitivity of over 80% in detecting breast cancer on recombined images [25,26].

Regarding reporting time, in our study, the shortest reporting time for MMG and DBT was 51 s and the longest was 186 s, with a median of 96 s per report, which was statistically higher than the reporting time for CEM, with the shortest reporting time of only 15 s and the longest of 183 s, with a median of 66 s per report. Assuming that the median reporting times are representative of typical cases, a radiologist could theoretically analyze approximately 54 CEM per hour compared to approximately 37 MMG and DBT per hour. Considering an 8 h workday, a radiologist could analyze 432 CEM and 296 MMG and DBT images. In practice, various factors such as case complexity, breaks, administrative tasks and variability in case difficulty will affect the actual number of cases reviewed per day. However, the comparison clearly shows the potential for a higher throughput with CEM.

This reduction in time not only improves workflow efficiency in clinical settings but also suggests that CEM can facilitate quicker diagnostic and treatment decisions, benefiting patient care. However, similar worst-case reporting times indicate that in the most complex cases, where detailed analysis is necessary, the time required to analyze and interpret the images may converge for both modalities. While CEM provides additional diagnostic information through contrast enhancement, it also requires the interpretation of both low-energy and high-energy images, which might be time-consuming in complex cases. Similarly, MMG, especially combined with DBT, involves reviewing multiple slices and projections, increasing the time required for thorough analysis. The similar worst-case reporting times also underscore the need for high diagnostic confidence in challenging breast imaging cases, necessitating careful review by radiologists. The study’s excellent interobserver agreement for CEM (ICC: 0.98) suggests that despite the longer time required, radiologists can reliably interpret CEM images.

These results are consistent with several previous studies. Patel et al. described the reading time for CEM as 60 to 120 s [27,28], while the suggested time for MMG is between 120 and 180 s (for MMG and DBT) [29]. Bernardi et al. evaluated the reading time for MMG and DBT in a screening setting and concluded that it averages 77 s per report (range 60–90 s) [30]. Another study by Dang estimated the mean interpretation time for the combined MMG and DBT to be 2.8 min ± 0.9 (range 1.5–4.2 min) [31].

In a study conducted by Savaridas et al., the estimated average reporting time for CEM was 3.65 min per study (range: 0.75–10 min), with the CEM reports prepared by 12 radiologists with varying levels of experience in reading CEM [32]. The average time in this study is slightly longer, as all CEM examinations were performed for preoperative assessment of newly diagnosed breast cancer.

In comparison, the reporting time for MRI is even longer, i.e., screening a complete protocol for breast MRI takes between 1 and 7 min, although the time for evaluation often varies according to complexity, with more like 15 min required to assess the features of just a single lesion in the preoperative setting [29,33]. In a study by Savaridas et al., the mean estimated reporting time for MRI was 20.63 min (range: 10–45 min) [32], compared with 3.65 min per CEM study (range: 0.75–10 min).

Regarding patient preparation and image acquisition time, CEM requires more preparation time compared to MMG and DBT, primarily due to the need for contrast media administration and monitoring. Preparation for CEM involves additional steps, including informing the patient about the examination protocol and potential side effects of the iodine-containing contrast media, obtaining consent, signing institutional consent forms, assessing renal function, placing an intravenous catheter in the antecubital fossa by a radiologist and administering contrast media. This preparation can take 15–20 min. Positioning and imaging are relatively quick, taking 8–10 min [28]. 

On the contrary, MMG and DBT require minimal preparation. Since they do not necessitate the administration of contrast media, the preparation time is significantly reduced, primarily focusing on explaining the procedure. The imaging process involves positioning and making multiple images to generate 3D image data of the classical CC and MLO projections of both breasts. Each image acquisition is quick (4–15 s), but the overall time is slightly longer due to the need for multiple images (3–5 min) [30].

When considering preparation, positioning and imaging times, CEM is more time-consuming (23–30 min) compared to MMG and DBT (10–15 min). However, despite the longer total time, CEM offers significant advantages in terms of diagnostic accuracy, sensitivity and specificity, making it a valuable imaging modality. The improved diagnostic confidence and reduced need for follow-up procedures may offset the additional time required. Effective workflow management and patient scheduling can help mitigate the impact of the longer procedure time, ensuring that the potential diagnostic advantages of CEM are maximized without significantly disrupting clinical workflow.

There are some limitations to our study. First, it is a single-center study, which may limit the generalizability of the findings. The specific patient population and clinical practices at our center might not be representative of other findings, potentially affecting the applicability of the results to broader populations. Second, the study design is retrospective, which inherently includes limitations such as selection bias and the inability to control all confounding variables. Prospective studies are needed to confirm these findings in a more controlled manner. Third, although CEM was compared with MMG and DBT, the study did not include direct comparison with MRI which is currently considered the gold standard for breast cancer imaging. Including MRI would have provided a more comprehensive evaluation of CEM’s diagnostic performance and accuracy. Fourth, the study does not provide long-term follow-up data on patient outcomes after imaging with CEM, MMG and DBT. Longitudinal studies would be necessary to assess the long-term effectiveness and potential benefits of CEM in clinical practice. 

Future research should focus on integrating CEM into standard screening protocols, conducting comparative studies with MRI, assessing long-term patient outcomes, exploring technological advancements in image processing and artificial intelligence (AI) for further reduction in reporting times as well as conducting patient-centered research to fully realize the potential of CEM in clinical practice.

## 5. Conclusions

Contrast-enhanced mammography is a new technique for the detection and diagnosis of breast cancer that is comparable in sensitivity and specificity to contrast-enhanced MRI but is more readily available, less expensive, easier to implement, quicker to learn and better tolerated by patients. This study aimed to validate the potential additional value of CEM in the field of breast imaging. It provided a robust comparison with standard MMG and DBT, demonstrating superior diagnostic accuracy, interobserver agreement and efficiency. The findings underscore CEM’s potential to enhance clinical practice by offering a reliable, quicker and more accurate imaging modality, particularly beneficial for patients with dense breast tissue or contraindications for MRI.

## Figures and Tables

**Table 1:** Demographic and clinical characteristics of participants.

All (N) = 316	Median	Range (min–max)	
Age	64	37–88	
	Number of participants		
	N	%	*p*-Value
Gender			
female	316	100	-
male	0	0	
History of previous breast biopsy/surgery			
yes	82	26	<0.001
no	234	74	
Recommended breast biopsy			
yes	89	28	<0.001
no	227	72	

**Table 2:** Interclass correlation coefficient between two breast radiologists (researcher 1 and researcher 2).

Researchers 1 and 2	ICC	95% CI
BI-RADS for MMG and DBT	0.73	0.66 to 0.78
BI-RADS for CEM	0.98	0.97 to 0.98

BI-RADS, Breast Imaging Reporting and Data System; MMG, mammography; DBT, digital breast tomosynthesis; CEM, contrast-enhanced mammography; ICC, interclass correlation coefficient; CI, confidence interval.

**Table 3:** Frequencies and differences of BI-RADS categories for MMG and DBT and CEM.

Examination	MMG and DBT	CEM	
	N (%)	N (%)	*p*-Value
BI-RADS			
0	165 (52.2)	0	-
1	42 (13.3)	77 (24.4)	0.153
2	7 (2.2)	125 (39.6)	0.048
3	0	0	-
4	47 (14.9)	45 (14.2)	0.927
5	55 (17.4)	69 (21.8)	0.543
6	0	0	-
Total	316 (100)	316 (100)	

BI-RADS, Breast Imaging Reporting and Data System; MMG, mammography; DBT, digital breast tomosynthesis; CEM, contrast-enhanced mammography.

**Table 4:** The reporting time for MMG and DBT and CEM.

Reporting Time in Seconds	MMG and DBT	CEM
Sample size	316	316
Lowest value	51	15
Highest value	186	183
Median	96	66
95% CI for the median	93–99	63–72
Z	−12.17	
*p*	<0.0001	

MMG, mammography; DBT, digital breast tomosynthesis; CEM, contrast-enhanced mammography; CI, confidence interval.

## Data Availability

All data generated or analyzed during this study are included in this article. Further enquiries can be directed to the corresponding author.
